# A barcode of multilocus nuclear DNA identifies genetic relatedness in pre- and post-Artemether/Lumefantrine treated *Plasmodium falciparum* in Nigeria

**DOI:** 10.1186/s12879-018-3314-3

**Published:** 2018-08-13

**Authors:** Kolapo Muyiwa Oyebola, Oluwagbemiga Olanrewaju Aina, Emmanuel Taiwo Idowu, Yetunde Adeola Olukosi, Olusola Sunday Ajibaye, Olubunmi Adetoro Otubanjo, Taiwo Samson Awolola, Gordon Akanzuwine Awandare, Alfred Amambua-Ngwa

**Affiliations:** 1 Medical Research Council Unit of The London School of Hygiene and Tropical Medicine, Bakau, The Gambia; 2 Parasitology and Bioinformatics, Department of Zoology, Faculty of Science, University of Lagos, Lagos, Nigeria; 3 West African Centre for Cell Biology of Infectious Pathogens, College of Basic and Applied Sciences, University of Ghana, Legon, Accra Ghana; 4 Malaria Research Group, Nigerian Institute of Medical Research, Lagos, Nigeria

**Keywords:** Artemisinin-based combination, Drug resistance, Parasite clearance, SNP barcode, Residual parasitaemia, Genetic relatedness

## Abstract

**Background:**

The decline in the efficacy of artemisinin-based combination treatment (ACT) in some endemic regions threatens the progress towards global elimination of malaria. Molecular surveillance of drug resistance in malaria-endemic regions is vital to detect the emergence and spread of mutant strains.

**Methods:**

We observed 89 malaria patients for the efficacy of artemether-lumefantrine for the treatment of uncomplicated *Plasmodium falciparum* infections in Lagos, Nigeria and determined the prevalence of drug resistant strains in the population. Parasite clearance rates were determined by microscopy and the highly sensitive *var* gene acidic terminal sequence (*var*ATS) polymerase chain reaction for 65 patients with samples on days 0, 1, 3, 7, 14, 21 and 28 after commencement of treatment. The genomic finger print of parasite DNA from pre- and post-treatment samples were determined using 24 nuclear single nucleotide polymorphisms (SNP) barcode for *P. falciparum*. Drug resistance associated alleles in chloroquine resistance transporter gene *(crt*-76), multidrug resistance genes *(mdr*1–86 and *mdr*1–184), dihydropteroate synthase (*dhps*-540), dihydrofolate reductase (*dhfr*-108) and kelch domain *(K*-*13*580) were genotyped by high resolution melt analysis of polymerase chain reaction (PCR) fragments.

**Results:**

By varATS qPCR, 12 (18.5%) of the participants had detectable parasite DNA in their blood three days after treatment, while eight (12.3%) individuals presented with genotypable day 28 parasitaemia. Complexity of infection (CoI) was 1.30 on day 0 and 1.34 on day 28, the mean expected heterozygosity (H_E_) values across all barcodes were 0.50 ± 0.05 and 0.56 ± 0.05 on days 0 and 28 respectively. Barcode (π) pairwise comparisons showed high genetic relatedness of day 0 and day 28 parasite isolates in three (37.5%) of the eight individuals who presented with re-appearing infections. *Crt*-76 mutant allele was present in 38 (58.5%) isolates. The *mdr*1–86 mutant allele was found in 56 (86.2%) isolates. No mutation in the *K*-*13*580 was observed.

**Conclusions:**

Persistence of DNA-detectable parasitaemia in more than 18% of cases after treatment and indications of genetic relatedness between pre- and post-treatment infections warrants further investigation of a larger population for signs of reduced ACT efficacy in Nigeria.

**Electronic supplementary material:**

The online version of this article (10.1186/s12879-018-3314-3) contains supplementary material, which is available to authorized users.

## Background

Malaria caused by *Plasmodium falciparum* infection is a significant public health problem in Nigeria where over 30% of the global burden of the disease is borne despite recent mass scale-up of intervention measures [1]. Artemisinin-based combination therapy (ACT) is recommended as first line treatment against uncomplicated malaria and it remains a critical tool in malaria control programmes. The most commonly used ACT, Artemether-Lumefantrine (AL), remains largely efficacious in sub-Saharan Africa [1]. The efficacy of AL has been attributed to the fast action of artemisinin and the resultant rapid reduction in the density of asexual malaria parasites, while lumefantrine acts to prevent recrudescence [2]. However, resistance to artemisinin-based drugs has been reported in many endemic regions in South-East Asia [3]. Although evidence of resistance among African populations of *Plasmodium falciparum* remains scarce, there have been emerging cases of potentially adaptive strains and slow parasite clearance following treatment [4, 5]. These parasites are often below the threshold of detection and are rarely re-treated, thus contributing to disease transmission and spread of resistance [6]. Accurate detection, quantification and characterization of such parasite isolates after treatment is necessary for the proper evaluation and monitoring of drug efficacy.

One recent technique for the ultra-sensitive detection of low parasitaemia involves the amplification of the *var* gene family present in the sub-telomere of the parasite [7]. Each parasite isolate comprises about 50–150 *var* genes [8], which possess acidic terminal sequence (ATS) with well-conserved domains that are targeted. Once detected, parasite isolates before and after treatment can be genetically fingerprinted with a single nucleotide polymorphism (SNP) barcode, which is another sensitive assay that requires only a small amount of input DNA and can be used to distinguish recrudescence from re-infection [9]. Prompt detection of mutations in loci implicated in drug resistance such as multidrug resistance gene (*mdr1*), chloroquine resistance transporter (*crt*), dihydrofolate reductase (*dhfr*), dihydropteroate synthase *(dhps*) and kelch-13 (*K13*) genes provides molecular evidence of antimalarial resistant parasites [10, 11]. For accurate surveillance of ACT resistance in a malaria-endemic setting as Nigeria, there is a need for continuous therapeutic efficacy studies using sensitive techniques that enable early detection of tolerant parasite strains. In this study, we assessed the efficacy of artemether-lumefantrine, determined genetic relatedness of parasites isolated before and after treatment and the prevalence of markers of drug resistance in the parasite population.

## Methods

### Study design and sample collection

**Fig. 1:**
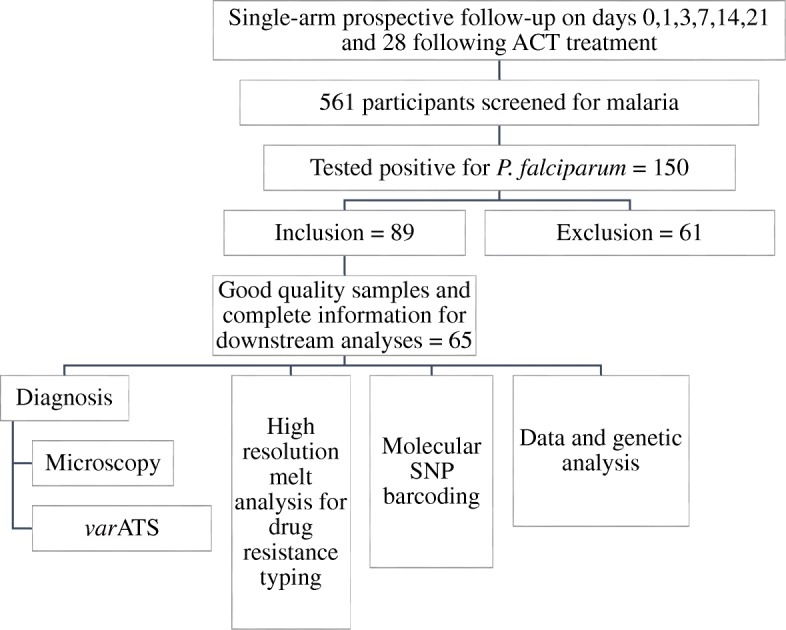
Study flow chart

This observational study of a 28-day follow-up was conducted in Ijede and Agbowa General Hospitals (GH) in Ikorodu, a peri-urban settlement in Lagos, South-West Nigeria, from August – November, 2016 following the revised WHO drug efficacy protocol [12]. Ijede GH is one of the sentinel sites used by the country’s Federal Ministry of Health (FMoH) for antimalarial drug efficacy trials. Individuals (1–70 years) presenting at the hospital with symptoms of uncomplicated malaria were screened for *Plasmodium falciparum*, first by rapid diagnostic test (RDT), followed by microscopy (Olympus BX53M, UK). The recruitment criteria included fever in the preceding two days, no history of antimalarial intake in the previous four weeks and *P. falciparum* parasitaemia > 2000/μl of blood on presentation [12]. Individuals with signs of complicated malaria or mixed infections were censored and those that developed concurrent ailments and complications during follow-up were promptly attended to by clinicians and hospital staff and therefore withdrawn from the study. Confirmed cases of uncomplicated malaria were treated with appropriate doses of AL (Coartem, Novartis, Switzerland) according to national policy on malaria treatment [13]. Based on an expected maximum treatment failure rate of 5% in a related population [14] and the desired confidence level (95%) and precision (5%), a minimum of 73 patients were required [12]. An additional 20% was added as provision for patients lost to follow up. Participants and/or their parents/guardians were instructed on how and when to take the evening dose. They were also advised to take fatty meals before or after drug intake to ensure proper absorption of the drug. Following consent, participants were followed up on days 1, 3, 7, 14, 21 and 28 post-treatment (Fig. 1). Confirmation of drug administration at home was done during the follow-up visits by tablet count. Patients were advised to return on any day during the follow-up period if symptoms returned and not to wait for our next scheduled visit day.

The primary endpoint was clinical and parasitological response within 28 days of commencement of treatment. The secondary endpoint was prevalence of drug resistance markers and genetic relatedness of pre- and post-treatment infections. Participants failing treatment, defined as the reappearance of parasitaemia after an initial complete clearance or failure of complete clearance on day 28, were retreated with AL whether or not they developed symptoms and after samples had been obtained for microscopy and molecular analyses. Before the first treatment was administered, dried blood spots from finger pricks were collected from individuals who gave informed consent and/or assent and also met the recruitment criteria.

### *var*ATS qPCR assessment of antimalarial treatment

*Plasmodium falciparum* DNA was extracted from pre- and post-treatment dried blood spots using the QiaAmp DNA minikit (Qiagen, Germany). The *var* gene acidic terminal sequence (*var*ATS) quantitative PCR was used to detect multi-copy genomic sequences of low-density infections in follow-up samples [7]. The primer/probe sequences and the cycling conditions are described in Additional file 1: Table S1. Briefly, 1 μl of PCR water, 10 μl of 2× Taqman Universal PCR Mastermix (Applied Biosystems, New Jersey, USA), 1.6 μl of 10uM forward and reverse primers, 0.8 μl of 10 μM probe and 5 μl of parasite DNA were vortexed and run on CFX 96 Touch™ Real-Time System (Bio-Rad Laboratories, CA, USA).

### Genotyping of antimalarial drug resistance SNPs

The prevalence of antimalarial drug resistance markers was determined using High Resolution Melt analysis of PCR fragments and probes targeting SNPs [15]. Six SNPs marking variant codons for antimalarial drug resistance were analysed: Chloroquine resistance transporter (*crt*-76), multidrug resistance *(mdr*1–86 an*d mdr*1–184), dihydropteroate synthase (*dhps*-540), dihydrofolate reductase (*dhfr*-108) and Kelch-13 (*K*-*13*580), (see Additional file 1: Table S2 for primer sequences and reaction conditions). Individual samples were traced through time points and the phenotype-genotype associations were profiled for day 0 and day 28 parasites. SNPs were genotyped for all samples positive for parasite DNA by PCR before treatment and on follow-up days.

### Molecular SNP barcoding

Molecular fingerprinting and changes in infection complexity before and after drug application was determined by SNP barcode assay. Day 0 and 28 parasitaemia barcodes were compared to determine if there was failure to clear the original parasites by the drug (recrudescence) or re-infection by other parasite strains. A panel of twenty-four unlinked neutral SNP barcodes with minor allele frequencies (MAF) ranging between 0.2–0.5 and representatively spread across the parasite genome (Additional file 1: Table S3) were used. Assays were performed as described by Daniels et al. [9], using the same primer and probe sequences. For pre- and post-treatment parasites, pairwise analysis, barcodes with a mixed allele call for more than one locus were considered as polygenomic and omitted from the analysis. Samples missing allele calls for more than five SNPs were also eliminated from the analysis.

### Data and genetic analyses

GraphPad Prism 7 (CA, USA) software was used for statistical analyses. Parasite clearance rates were determined by the Parasite Clearance Estimator (PCE) tool (http://www.wwarn.org). The varATS qPCR survival estimates of parasites at different time-points were determined using Kaplan-Meier survival analysis. Association between age and parasite counts on days 0, 3 and 28 was assessed using Spearman’s correlation coefficient (r_s_). The relationships between enrollment parasite density on day 0 and parasitaemia on days 3 and 28 were also determined using Spearman’s correlation coefficient. Bonferroni correction was used to adjust multiple testing of age and parasitaemia on days 0, 3 and 28. To determine the genetic identity of each pair of samples on day 0 and day 28, we calculated pairwise barcode similarity index (π), counting ambiguous or missing calls as mismatches [16]. To avoid biasing the percent similarity because of matching major alleles, we limited the percent similarity calculations to include only sites where the minor alleles were present in pairwise barcodes of day 0 and day 28 isolates. The similarity index, π, was calculated as the number of sites where the minor allele matched divided by the total number of sites where the minor allele was present. π has a potential range from 0 (no genetic relatedness) to 1(high genetic relatedness). The allele frequencies per SNP barcode, allelic diversity in pre-treatment and day 28 parasite populations and allele frequencies per barcode per population were calculated using GENALEX 6 [17]. Complexity of infection per SNP barcode was estimated using COIL [18]. Allelic diversity was calculated for each of SNP barcodes based on the allele frequencies, using the formula Nei’s unbiased genetic diversity index for expected heterozygosity, H_E_ = [$\left(\frac{n}{n-1}\right)\left(1-\sum {P}^2\right)\Big]$, where n is the number of isolates analyzed and *P* represents the frequency of each allele of the SNP. H_E_ provides an indication of the probability that two samples will be different. It has a potential range from 0 (no allele diversity) to 1 (high allelic diversity). All statistical assumptions were made at 95% confidence limit.

## Results

### Baseline information on the study participants

In this study, 561 individuals were screened, 150 of them tested positive for falciparum malaria. Eighty-nine patients met the inclusion criteria, were enrolled and followed up. Sixty-five participants had complete sample information for days 1, 3, 7, 14, 21 and 28 after commencement of treatment and were used for genetic analyses. The demographic profiles of the participants are presented in Table 1. Participants were stratified into three age groups: 1-5 yrs.; 6–14 years and ≥ 15 years old (Table 1).

**Table 1:** Summary of participant characteristics at baseline

Characteristic		Number screened n (%)	Number positive for *P. falciparum* n (%)	Number enrolled n (%)
Sex	Male	157 (27.99)	19 (12.67)	18 (27.69)
	Female	404 (72.01)	131 (87.37)	47 (72.31)
Age group	1–5 years	138 (24.60)	22 (14.67)	13 (20.0)
	6–14 years	80 (14.26)	12 (8.0)	21 (32.31)
	≥15 years	343 (61.14)	116 (77.33)	31 (47.69)

### Parasite clearance rates

**Fig. 2:**
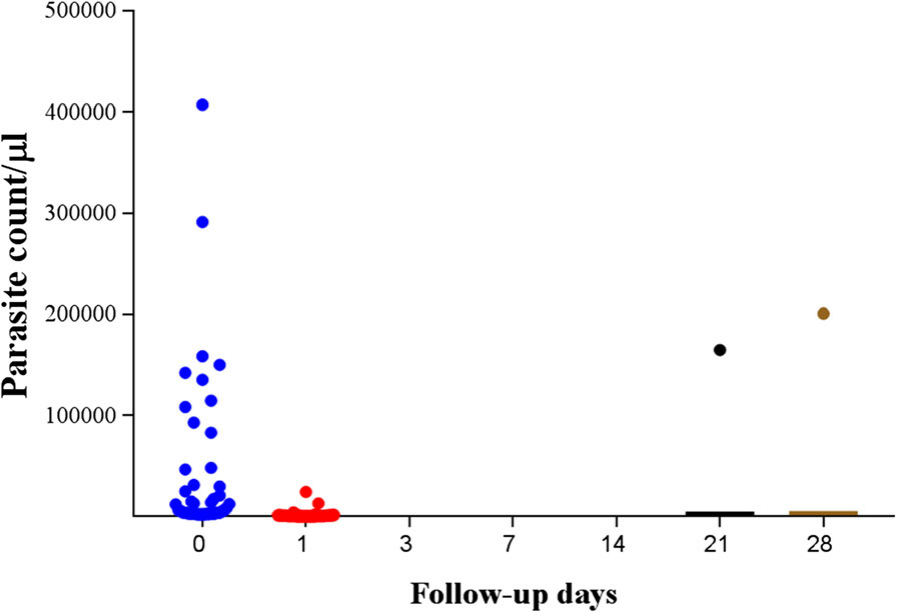
Parasite count/μl by microscopy before and after artemether/lumefantrine treatment. (After day 1, the mean concentration was the same up to day 14 with re-appearing infections on days 21 and 28)

**Fig. 3:**
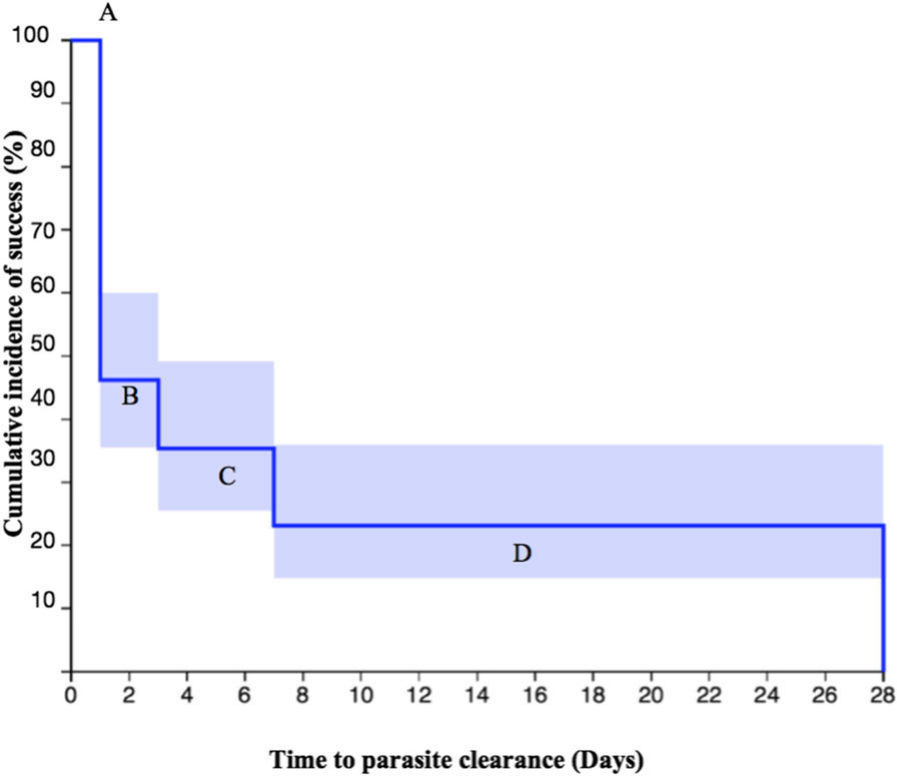
Kaplan-Meier estimates of parasite reduction following artemether-lumefantrine treatment (Group A represents % reduction of parasitaemia on day 1, (ii) Group B represents % reduction of parasitaemia on day 3, Group C represents % reduction of parasitaemia on day 7, and Group D represents % parasitaemia beyond day 7

**Fig. 4:**
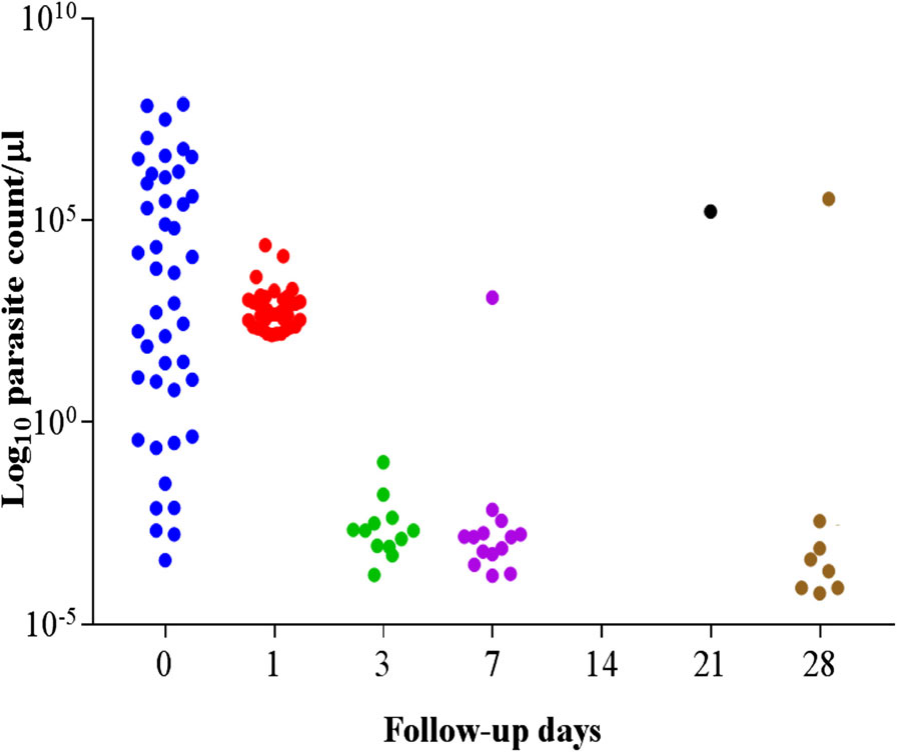
Log_10_ Parasite count/μl by varATS qPCR before and after artemether/lumefantrine treatment (Low-grade residual infections persist on days 1, 3, 7, 4 and 28)

Microscopic analysis showed that none of the participants presented with parasitaemia on day 3 while one of the participants presented with parasitaemia on day 28 (Fig. 2). Based on the detection of parasite DNA in peripheral blood by *var*ATS PCR assay, four main parasite clearance profiles could be identified (Fig. 3): Group A- individuals who cleared infection by day 1, (ii) Group B-those who remained positive until day 3, Group C-individuals who remained positive until day 7, and Group D-those who tested positive beyond day 7. *var*ATS results showed that 12 (18.5%) of participants presented with parasitaemia 3 days post-treatment while eight (12.3%) patients presented with genotypable day 28 parasitaemia (Fig. 4).

### Relationship between enrolment parasite density and post-treatment parasitaemia

The relationship between parasite persistence after treatment and the levels of parasitaemia at enrolment was examined by varATS qPCR using Spearman’s correlation coefficient (r_s_). There was no linear association (*P* = 0.79) between the starting parasite density and day 3 parasitaemia. Similarly, there was no correlation (*P* = 0.69) between the enrolment parasitaemia and day 28 parasite count. There was a weak linear association between parasite densities on day 3 and reappearance on day 28 (rs = 0.12) even though the relationship was not statistically significant (*P* = 0.33). Analysis of parasitaemia and patient characteristics (Table 2) did not indicate any relationship between age of the participant and parasite density on day 0 (*P* = 0.25). There was a weak inverse association between the age of patients and (i) persistence of parasitaemia on day 3 (r_s_ = − 0.24; *P* = 0.056) and (ii) re-appearance of parasitaemia on day 28 (rs = − 0.41; *P* = 0.0008). After adjusting for multiple testing using Bonferroni method and with a new cut-off of 0.0167 for the tests between age and days 0, 3 and 28 parasitaemia, we observed an inverse relationship between parasitaemia on day 28 and age of the patient. None of the patients with positive parasitaemia post-treatment reported with clinical symptoms of malaria.

**Table 2:** Mean (and standard deviation) of parasitaemia (by varATS) per age group

Age group	Day 0	Day 1	Day 3	Day 7	Day 28
1–5 years	7,856,186.72 ± 19,107,401.41	89,555.78 ± 198,852.33	0.00172 ± 0.00433	0.00047 ± 0.00098	17,651.29 ± 59,342.22
6–14 years	5,365,728.80 ± 18,300,622.30	2640.90 ± 2553.02	0.00051 ± 0.00110	0.00057 ± 0.00035	11,157.18 ± 11,157.14
≥15 years	519,906.10 ± 1,394,260.12	3835.63 ± 1704.04	0.00340 ± 0.01810	41.50 ± 41.50	0.00015 ± 0.00079

### Genetic description of day 0 and day 28 parasite populations

Allele frequencies at each of the barcodes in the day 0 and day 28 populations are presented in Additional file 2: Datasheet S1. The complexity of infection in the day 0 population was 1.30 while the CoI on day 28 marginally increased to 1.34 on day 28 (Additional file 2: Datasheet S2), however, this difference was not statistically significant (*P* = 0.42). The mean H_E_ values across all barcodes on day 0 was 0.50 ± 0.05 and 0.56 ± 0.05 on day 28. There was no evidence of significant linkage disequilibrium (Additional file 1: Table S4), although the ${I}_A^S$ value was higher in day 0 (0.08) than in day 28 populations (0.03).

### Signatures of relatedness between individual pre- and post-treatment infections

We used the analysis of barcode sequence identity to estimate relatedness between pairwise parasite isolates in day 0 and day 28 infections (Additional file 2: Datasheet S3). The barcode (π) pairwise comparisons showed high genetic relatedness of day 0 and re-appearing day 28 parasite isolates in three (37.5%) of the eight participants who presented with day 28 parasitaemia (Table 3).

**Table 3:** Age of participants and barcode similarity (π) indices of parasites before and after drug treatment

Sample ID	Age (years)	^a^π
IJD 244	2	0.23
IJD 254	10	0.30
IJD 095	10	0.36
IJD 393	3	0.47
AJ 008	14	0.59
IJD 203	7	0.83
IJD 253	4	0.94
AGB 002	3	1.0

^a^Barcode similarity index (π) close to 1 suggests high genetic relatedness between two parasite isolates

### Prevalence of drug resistance polymorphisms

**Fig. 5:**
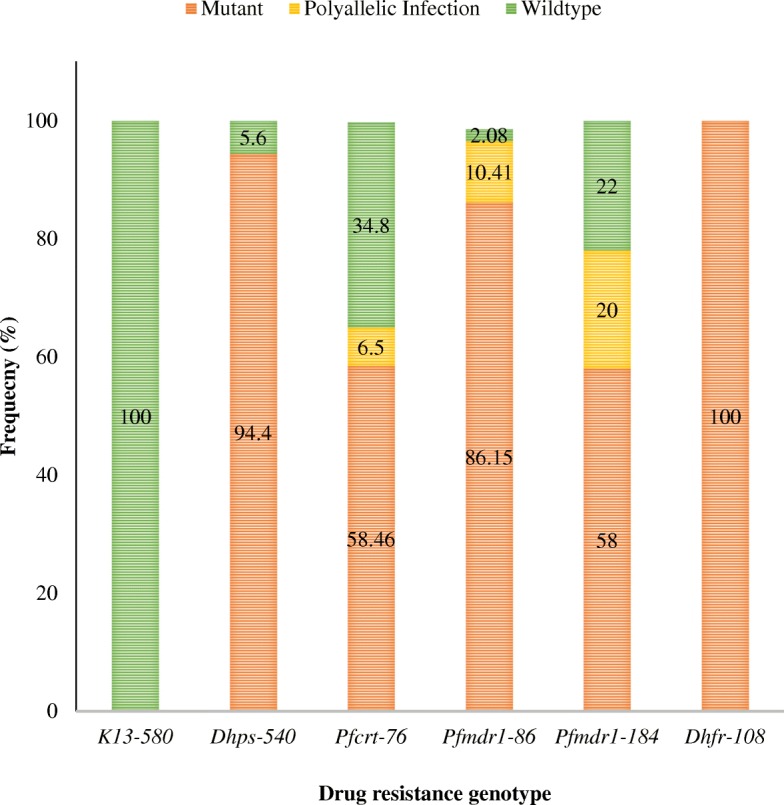
Prevalence of polymorphisms in *Plasmodium falciparum* drug resistance genes in pre-treatment parasite population (*K13*: Kelch 13 gene; *Dhps*: Dihydropteroate synthase; *Pfcrt*: *Plasmodium falciparum* chloroquine resistance transporter gene; *Pfmdr1*: *Plasmodium falciparum* chloroquine resistance transporter gene; *Dhfr*: Dihydrofolate reductase)

When the pre-treatment parasites were taken together, the *crt*-76 T mutant allele was present in 38 (58.5%) isolates. The *mdr*1–86 mutant allele was found in 56 (86.2%) of isolates. We observed 94.4% mutant and 5.6% wildtype alleles of *Dhps*-540 and 100% mutant alleles of *Dhfr*-108 in the population*.* No mutant allele of the *K*-*13* C580Y was observed (Fig. 5). Pairwise phenotype-genotype comparisons showed no association of *mdr*1 (N86Y and Y184F) and *crt* K76 T mutations with persistence of parasitaemia on day 3 (Table 4) and re-appearance on day 28 (Table 5).

**Table 4:** Phenotype-genotype association of infections on day 3

	Genotype n (%)											
	*mdr1*–86			*mdr1*–184			*crt*-76			*K13*–580		
Phenotype	N	Y	N/Y	Y	F	Y/F	K	T	K/T	C	Y	C/Y
Individuals with parasites on day 3 = 12	0 (0%)	10 (83.3%)	2 (16.7%)	2 (14.3%)	8 (66.6%)	2 (16.7%)	2 (16.7%)	8(66.6%)	2 (16.7%)	12 (100%)	0 (0%)	0 (0%)
Individuals with full clearance of parasites on day 3 = 53	5 (9.43%)	46 (86.8%)	2 (3.8%)	11 (20.8%)	29 (54.7%)	13 (24.5%)	10 (18.9	40 (75.5%)	3 (5.7%)	53 (100%)	0 (0%)	0 (0%)

N86 = mdr1–86 wildtype allele; 86Y = mdr1–86 mutant allele; Y184 = mdr1–184 wildtype allele; 184F = mdr1–184 mutant allele; K76 = crt-76 wildtype allele; 76 T = crt-76 mutant allele; C580 = K13 wildtype allele; 580Y = K13 mutant allele

**Table 5:** Pairwise comparison of drug resistance genotypes in pre-treatment and re-appearing *P. falciparum* infections

Sample ID	*mdr*1-N86Y		*mdr*1-Y184F		*crt*-K76 T		*K13*-C580Y	
	Day 0	Day 28	Day 0	Day 28	Day 0	Day 28	Day 0	Day 28
IJD 244	Y	Y	F	F	T	T	C	C
IJD 254	Y	Y	F	F	T	T	C	C
IJD 095	Y	Y	F	F	T	T	C	C
IJD 393	N	N	F	F	T	T	C	C
AJ 008	Y	Y	F	F	T	T	C	C
IJD 203	Y	Y	Y/F	F	T	T	C	C
IJD 253	Y	Y	F	F	T	T	C	C
AGB 002	N	N	F	F	K	K	C	C

N86 = mdr1–86 wildtype allele; 86Y = mdr1–86 mutant allele; Y184 = mdr1–184 wildtype allele; 184F = mdr1–184 mutant allele; K76 = crt-76 wildtype allele; 76 T = crt-76 mutant allele; C580 = K13 wildtype allele; 580Y = K13 mutant allele

## Discussion

One of the key approaches to malaria control is prompt treatment of patients for an improvement in health and prevention of onward transmission. With rising reports of drug-resistant *Plasmodium falciparum*, surveillance of ACT efficacy becomes important so that recrudescent infections can be promptly detected, treated and documented. In this study, we reported in vivo re-appearance of parasites following appropriate treatment of uncomplicated *P. falciparum* with artemether-lumefantrine in Nigeria. The genomic barcode analysis of pre-treatment and re-appearing infections suggested recrudescence despite the absence of mutation in K-13 C580Y. This suggests that the potency of the most promising antimalarial drug, artemisinin-based combination, in treating *P. falciparum* infection in Nigeria may be progressively waning.

High genetic diversity of the parasites was observed in the population. This observation is in line with expectations from high transmission settings where multiple parasite genomes in each sample and highly variable combinations of barcodes among individuals in the population have been observed [19]. We also observed a marginal increase in CoI 28 days after treatment suggesting a possible lack of clonal expansion following treatment. This agrees with the observation in Kenya where, contrary to expectations of reduced CoIs caused by the persistence of only drug-resistant strains in post-treatment infections, CoI on day 28 was higher than day 0 [20].

Age and immunity have previously been demonstrated as indicators of varying therapeutic responses [21]. Thus, older individuals living in a high transmission area who have a more developed acquired immunity than children in the same setting would be expected to have fewer persistent infections following treatment. While our finding could not directly correlate age with the persistence of parasitaemia on the third day of treatment, it revealed re-appearance of infections on day 28 only among children less than fifteen years old. This observation possibly explains the role of acquired immunity in complementing antimalarial drug action in parasite clearance. However, additional studies are required to understand the influence of immunity on therapeutic response and drug resistance by correlating parasite clearance rates with malaria antibody titres in the individuals.

Although we did not observe any clinical outcome of persistent infections and there was no direct association between re-appearance of infection and day 0 parasite densities, it is important to continue monitoring the prevalence of residual infections following treatment, even though they were observed in low quantities in this study. Since parasitaemia as low as 0.5/μl of blood corresponds to as high as approximately one million parasites in the body [21], low-grade infections observed after treatment in this study area may sustain a reservoir for silent transmission of the disease. Studies that involve longer follow-up durations and laboratory adaptation of the sub-microscopic parasites may provide more information on the transmission potential of these post-treatment parasites.

A high frequency of mutant alleles of *crt* and *mdr*1 (N86Y and Y134F) was reported in this study, but these mutations have not been directly associated with post- treatment re-appearance of infections. In addition, contrary to a previous report showing that polymorphisms in *mdr1* genes were associated with decreased sensitivity to lumefantrine [22], our phenotype-genotype association study provided no evidence that the presence of 86Y allele sensitized parasites to lumefantrine as there was no correlation between the distribution of N86 and 134F alleles and presence of parasites on day 28. An increase in the frequency of *Pfcrt* 76 T is associated with reduced sensitivity to chloroquine [23] and the removal of drug pressure is expected to cause a decline in resistance conferring mutations [24]. The prevalence of mutant 76 T allele in this present investigation is lower than previously reported before the proscription of CQ for mild malaria treatment in Nigeria [25]. This may be a pointer to ongoing selection for chloroquine-susceptible parasites following widespread use of artemisinin. Kelch-13 sequencing information from recent therapeutic efficacy studies conducted in Cambodia, Lao and Vietnam has revealed cysteine to tyrosine mutation in codon 580 of K13 gene (C580Y) as the dominant polymorphism associated with artemisinin resistance [3]. However, our investigation revealed the absence of this mutation in re-appearing parasites.

We propose further investigations involving complete genome-wide associations between pre-treatment and persistent parasites to circumvent the limitation of our study which did not preclude the influence of re-infection with the same parasite type or residual parasite DNA and non-replicating mature gametocytes that can persist at low concentrations after treatment. Computational analyses that involve the combination of genomic information with epidemiological simulations will present complementary tools for interpreting population-level impact of drug interventions. In addition, since *P. falciparum* is thought to exhibit drug-induced developmental arrest until the activity of artemisinin in the host has diminished [26], details about quiescence in post-treatment infections using in vitro models may further explain the underlying mechanisms of re-appearance of parasites after treatment in the study area. A broader line of investigation on the efficacy of ACTs in larger populations in the country and across the borders is necessary to understand spatial and temporal heterogeneity of infectious reservoirs post-treatment.

## Conclusions

We have reported in vivo re-appearance of parasites following appropriate treatment of uncomplicated *Plasmodium falciparum* with Artemether/Lumefantrine (AL) in Nigeria. The genomic barcode analysis of pre-treatment and re-appearing infections suggested recrudescence despite the absence of mutation in K-13 C580Y. This finding implies that the Nigerian parasites could be deploying a more complex drug evasion mechanism beyond the C580Y polymorphism. Further investigation of a larger population of parasites in the country is required for signs of reduced efficacy of artemisinin-based treatment of malaria.

## Additional files

Additional file 1: **Table S1.** Primer sequences and qPCR conditions for *var*ATS assay. **Table S2.** High Resolution Melting Drug Resistance Assay. Table 3 Relationship between day 0 parasite density and percentage of individuals with day 3 parasitaemia. **Table S4.** Linkage disequilibrium analysis for pre- and post-treatment parasite populations. (DOCX 26 kb)

Additional file 2: **Datasheet S1.** Allele frequencies at each of the barcodes in the day 0 and day 28 populations. **Datasheet S2.** Complexity of infection in day 0 and day 28 populations. **Datasheet S3.** Pairwise sequence comparison of day 0 and day 28 parasite isolates. (ZIP 94 kb)

## Abbreviations

ACT: Artemisinin-based Combination Treatment
AL: Artemether/Lumefantrine
CoI: Complexity of Infection
*crt*: Chloroquine Resistance Transporter
*dhfr*: Dihydrofolate Reductase
*dhps*: Dihydropteroate Synthase
H_E_: Expected Heterozygosity
HRM: High Resolution Melting
K-13: Kelch-13 Domain
MAF: Minor Allele Frequency
*mdr*: Multidrug Resistance
PCE: Parasite Clearance Estimator
PCR: Polymerase Chain Reaction
RDT: Rapid Diagnostic Test
SNP: Single Nucleotide Polymorphism
*var*ATS: *var* gene Acidic Terminal Sequence
