# Supplementary material for: A barcode of multilocus nuclear DNA identifies genetic relatedness in pre- and post-Artemether/Lumefantrine treated Plasmodium falciparum in Nigeria
Source: BMC Infect Dis. 2018 Aug 13;18:392. doi: 10.1186/s12879-018-3314-3 (PMC6205152; doi:10.1186/s12879-018-3314-3)
Supplement: Supplementary file 1 — Table S1. Primer sequences and qPCR conditions for varATS assay. Table S2. High Resolution Melting Drug Resistance Assay. Table 3 Relationship between day 0 parasite density and percentage of individuals with day 3 parasitaemia. Table S4. Linkage disequilibrium analysis for pre- and post-treatment parasite populations. (DOCX 26 kb) [file 12879_2018_3314_MOESM1_ESM.docx]

**Table S1 Primer sequences and qPCR conditions for *var*ATS assay**

| Oligonucleotide sequences | |
| --- | --- |
| Primer-fw (5’-3’) | CCCATACACAACCAATTGGA |
| Primer-rev (5’-3’) | TTCGCACATATCTCTATGTCTATCT |
| Probe (5’-3’) | 6-FAM-TTTTCCATAAATGGT-NFQ-MGB |
| qPCR reaction conditions (final concentration in qPCR mix) | |
| Total volume (μl) | 20 |
| DNA volume (μl) | 5 |
| TaqMan^®^ Gene Expression Mastermix | 1x |
| Primer (each) | 800 nM |
| Probe | 400 nM |
| qPCR cycling conditions | |
| Pre-incubation | 2 min – 50°C |
| Initial denaturation | 10 min – 95°C |
| Denaturation | 15 sec – 95 °C |
| Annealing & Elongation | 1 min – 55°C |
| Number of cycles | 45 |
| Standard material for quantification | gDNA of parasite dilution row |
| Platform | Real-Time PCR System (Applied Biosystems) |

The *var*ATS probe and all qPCR reagents were purchased from Applied Biosystems/Life Technologies.

**Table S2 High Resolution Melting Drug Resistance Assay**

| Oligonucleotide sequences | | | |  |
| --- | --- | --- | --- | --- |
| Primer-fw (5’-3’) | Pfcrt-76 |  | GTAAAACGACGGCCAGTTTCTTGTCTTGGTAAATGTGCTA |  |
|  | Pfmdr1-86 |  | TTATTATTTATATCATTTGTATGTGCTGTATTATCAGG |  |
|  | Pfmdr1-184 |  | AGTTCAGGAATTGGACGAAATTTATAACA |  |
|  | Dhps-540 |  | GTGTTGATAATGATTTAGTTGATATATTAAATGATATTAAATGATATTAGTGC |  |
|  | Dhfr-108 |  | CTGTGGATAATGTAAATGATATGCCTAATTCTA |  |
|  | PfK13-580 |  |  |  |
| Primer-rev (5’-3’) | Pfcrt-76 |  | CAGGAAACAGCTATGACCGGATGTTACAAAACTATAGTTACCAAT |  |
|  | Pfmdr1-86 |  | CAGGAAACAGCTATGACATCATTGATAATATAAATTGTACTAAACCTATAGATACT |  |
|  | Pfmdr1-184 |  | ACGCAAGTAATACATAAAGTCAAACG |  |
|  | Dhps-540 |  | GTTTATCCATTGTATGTGGATTTCCTCTT |  |
|  | Dhfr-108 |  | GACAATATAACATTTATCCTATTGCTTAAAGGT |  |
|  | PfK13-580 |  |  |  |
| Probe (5’-3’) |  |  |  | |
| qPCR reaction conditions (final concentration in qPCR mix) | | | | |
| Total volume (μl) | | 10 | | |
| DNA volume (μl) | | 5 | | |
| LightScanner Mastermix | | 2.5x | | |
| qPCR cycling conditions | | | | |
| Pre-incubation | | 2 min – 95°C | | |
| Initial denaturation | | 30 sec – 94°C | | |
| Denaturation | | 30 sec – 66 °C | | |
| Annealing & Elongation | | 30 sec – 74°C | | |
| Number of cycles | | 45 | | |
|  | | 30 sec – 94^0^C | | |
| Melt | | 40^0^C – 90^0^C (0.2^0^C/sec) | | |
| Platform | | LightScanner | | |

Table S3 Relationship between day o parasite density and percentage of individuals with day3 parasitaemia

| Parasite count/µl | Percentage of individuals positive on day 3 |
| --- | --- |
| 2,000-10,000 | 16.67 |
| 10,001-20,000 | 0 |
| 20,001-50,000 | 16.67 |
| 50,001-100,000 | 75 |
| 100,001-200,000 | 0 |
| 200,001-500,000 | 40 |
| 500,001-1000,000 | 16.67 |
